#  A Review of the Current Research Trends in the Application of Medicinal Plants as a Source for Novel Therapeutic Agents Against *Acanthamoeba *Infections

**Published:** 2016

**Authors:** Maryam Niyyati, Samira Dodangeh, Jacob Lorenzo-Morales

**Affiliations:** a *Department of Medical Parasitology and Mycology, School of Medicine, Shahid Beheshti University of Medical Sciences, Tehran, Iran. *; b *University Institute of Tropical Diseases and Public Health of the Canary Islands, University of La Laguna, Avda. AstrofísicoFco. Sánchez, S/N, 38203 La Laguna, Tenerife, Canary Islands, Spain.*

**Keywords:** Medicinal plant, *Acanthamoeba* infections, Chemical therapy

## Abstract

*Acanthamoeba* keratitis (AK) is a sight-threating infection of the cornea that mostly affects contact lens wearers. Until now, AK treatment remains very difficult due to the existence of a highly resistant cyst stage in the life cycle of *Acanthamoeba* which is extremely resistant to most of the available anti-amoebic compounds. Moreover, current treatment of AK is usually based in the combination of various therapeutic agents such as polyhexamethylene biguanide or chlorhexidine and propamidine isethionate. However, all the mentioned compounds have also showed toxic side effects on human keratocytes and presented poor cysticidal effect at the concentrations currently used in the established AK treatments.

Nowadays, the elucidation of novel compounds with antimicrobial and anticancer properties from plant and herbs with medicinal properties have encouraged researchers to evaluate plants as a source of new molecules with anti-trophozoite and cysticidal effects.

Thus, in recent years, many natural products have been reported to present potent anti-*Acanthamoeba* properties with good selectivity and minimal toxic effects. Therefore, the chemical drugs currently used for AK treatment, their drawbacks as well as the current research in medicinal plants as a source of potent anti-*Acanthamoeba* compounds are described in this review.

## Introduction

*Acanthamoeba* spp. are free-living amoebae with the potential of being opportunistic pathogens for humans and animals. There are two stages in their life cycle: an active trophozoite form and the double-walled highly resistant cyst. Trophozoites inhabit a variety of bacteria-contained niches such as fresh water bodies, hot springs, soil, drinking water, bottled water, dental treatment units, dialysis units, fluids of contact lenses and infected tissue cultures among others (Table 1.) (1). As mentioned before, the Cyst form of *Acanthamoeba *is highly resistant to a vast range of temperature, pH, and anti-microbial agents (2). Furthermore, this amoebic genus is the causative agent of two severe diseases in humans: *Acanthamoeba* keratitis which is serious corneal infection that can develop into blindness and usually reported in contact lens wearers, and the fatal Granulomatous Amoebic Encephalitis (GAE) which mostly affects immunocompromised individuals (3, 4). *Acanthamoeba* also may cause other diseases such as cutaneous ulcers, abscesses, arthritis, and/or rhinosinusitis (5).

GAE is a relatively rare disease. Clinical characteristics include headache, fever, nausea, vomiting, behavioral changes, stiff neck, lethargy and increased intracranial pressure. In later stages of the infection also symptoms such as loss of consciousness, seizures, coma, and death have been reported. Approximately more than 150 cases have been reported worldwide (6, 7).

*Acanthamoeba* keratitis (AK) usually manifests in the early stages of infection with inflammation, eye redness, epithelial defects and photophobia, edema and intense pain. Moreover, if it not diagnosed and treated on time, it may even end in blindness (8). Previous studies in the early to mid-1980 reported an exponential increase in the number of individuals infected with this amoeba (9). This is mainly due to an increased number of soft contact lens wearers and improper use and maintenance of the lenses. Furthermore, it is worthy to mention that 85% of AK cases are detected in soft contact lens wearers (10, 11). In a more recent study in 2007, AK reported case were estimated to be higher than 3000 (6). Therefore, it is clear that the number of AK reported cases continues to rise worldwide. 

*Methodology based on search strategy*

A systematic review based on database sources such as Medline, PubMed, Scopus and Google scholar was conducted in this study. No restrictions were placed on study date, design or language of publication including all valuable and relevant information containing the keywords *Acanthamoeba* and therapy. We also referred to the databases of Medline, PubMed, Scopus and Google scholar and the keywords *Acanthamoeba* and Amoebic Keratitis, and words including treatment, medicinal plants and herbal medicine. Furthermore, information in books associated to *Acanthamoeba* and treatment strategy was also included as well as abstracts and full articles that were written in English and showed to be relevant to the topic described above. Only reports and studies with minimal relevance were excluded from this study.

*Current therapy of Acanthamoeba infection*

*Chemical treatment and their drawback*

Effective treatment of CNS-related infections due to *Acanthamoeba* has been recorded as a combined treatment, normally started at an early stage of the infection. However, in the later stages of the infection, the majority of therapeutic agents were reported not to be effective (12). Overall, combination chemotherapies were found more successful than single-drug therapies, Therefore, usual therapeutic agents reported so far include a combination of drugs such as ketoconazole, fluconazole, itraconazole, pentamidine isethionate, azithromycin, sulfadiazine, amphotericin B, rifampicin, voriconazole and miltefosine (12). Because of ineffective therapy, GAE is often deadly, thus less than 10 GAE patients have recovered with the application of a combination ofthe drugs mentioned above (13). 

**Table 1:** Characteristics of *Acanthamoeba* spp. as an agents of amoebic encephalitis and amoebic keratitis (Visvesvara *et* *al*, 2007).

***Acanthamoeba *** **spp.**		
**Life cycle**	**Two forms: trophozoite and cyst**	
Morphological Features	Trophozoite: Vesicular nucleus; spine-like pseudopodia projecting from surface; cyst: wall with two layers	
*In vitro *cultivation	Axenic, bacterized, and defined media; tissue culture cells; growth at 37 °C (CNS isolates) or 30 °C (keratitis isolates)	
The most important diseases	**Granulomatous Amoebic** **Encephalitis (GAE)**	**Amoebic keratitis (AK)**
Incubation period	Weeks to months (GAE)	Days (AK)
High risk people	Typically Immune-compromised Individuals such as AIDs patients (GAE)	Mainly contact-lens wearers; Low secretory IgA may contribute (AK)
Clinical Characteristics	Headache, fever, nausea, vomiting, behavioral changes, stiff neck, lethargy, loss of consciousness, seizures, coma, and death (GAE)	Painful, sight- redness, photophobia, edema (AK)
Clinical course	Sub-acute course; acute stage fatal in weeks (GAE)	Penetration of amoebae into cornea; stromal ring due to PMN infiltrate (AK)
Laboratory diagnostic methods	Amoeba seen in CSF; Molecular method (GAE)	Not relevant (AK)
Neuroimaging (CT and/or MRI)	Presence of space occupying or ring enhancing lesion (GAE)	Corneal scrapings or biopsy; confocal microscopy; PCR (AK)
Prevention	Monitoring of environmental sources such as waters, ventilators, air conditioning units (GAE)	Use of anti-acanthamoeba lens solutions; avoiding swimming or bathing with contact lenses (AK)
Chemical therapy	Combination of drugs such as ketoconazole, fluconazole, itraconazole, azithromycin, sulfadiazine, amphotericin B, rifampin, voriconazole, and miltefosine (GAE)	Combination chemotherapeutic agents such as polyhexamethylene biguanide, chlorhexidine (AK)
Prognosis	Poor; diagnosis is often Post-mortem, only a few patients have survived (GAE)	Good with early diagnosis and proper treatment (AK)

**Table 2:** Several medicinal plants with reported activity against *Acanthamoeba* *spp*

**Plant**	**Extract**	**Effective** **Concentration (trophozoite)**	**Effect time** **(trophozoite)**	**Percentages** **of viable** **trophozoites**	**Effective** **Concentration (cyst)**	**Effective time** **(cyst)**	**Percentages of viable cysts**
***Thymus*** ***sipyleus subsp. Sipyleus var. Sipyleus***	Methanol	32 mg/mL	3 h	0	32 mg/mL	12 h	0
***Satureja*** ***cuneifolia***	Methanol	32 mg/mL	24 h	0	32 mg/mL	72 h	53/07
***Melissa*** ***officinalis***	Methanol	32 mg/mL	72 h	55/07	32 mg/ mL	72 h	70/0
***Trigonella*** ***foenum*** ***graecum***	Chloroformic	10 mg/mL	48 h	0	10 mg/mL	72 h	0
***Origanum syriacum***	Methanol	32 mg/mL	3 h	0	32 mg/mL	24 h	0
***Helianthemum*** ***lippii***	Ethyl acetate	N/A*	N/A*	N/A*	500 mg/mL	72 h	25
***Arachis hypogaea L***	Ethanol	N/A*	N/A*	N/A*	100 mg/mL (MIC**)	24 h	0
***Curcuma longa L***	Ethanol	N/A*	N/A*	N/A*	1 g/ml (MIC)	48 h	0
***Pancratium*** ***maritimum L***	Ethanol	N/A*	N/A*	N/A*	200 mg/mL (MIC)	72 h	0
***Inula oculus- christi (L)***	Aqueous	32 mg/mL	24 h	0	32 mg/mL	72 h	74/7

* N/A= Not Applicable

** MIC= Minimum Inhibitory Concentration.

Regarding, *Acanthamoeba* keratitis (AK) treatments reported so far, the combination of chemotherapeutic agents such as polyhexamethylene biguanide, which destroys cell membranes, and propamidine isethionate, which inhibits DNA synthesis (14, 15) is often used. Moreover, chlorhexidine, alone or in combination with other drugs, has also been applied for AK treatment (16, 17). Unfortunately, propamidine is poorly cysticidal and even resistance to this compound has been reported in *Acanthamoeba* strains (18, 19). 

In the case of a persistent infection with inflammation, corticosteroids may be used. However, their use is controversial because they cause suppression of the immunological response of the patient. Moreover, corticosteroids produce inhibition of the processes of encystation and excystation of *Acanthamoeba*, which could be a cause for the appearance of resistance problems (1). Recent studies have highlighted an association of topical corticosteroids and a diagnostic delay of AK (1, 15, 20). 

It is also important to mention that the described combination treatment are normally only active against the trophozoite stage and therefore, *Acanthamoeba* cysts could remain viable and lead to serious and frequent recurrences of keratitis. Moreover, resistance of the double walled cysts is mainly due to cellulose molecules presented in the inner layer of the cysts. In addition, the majority of drugs mentioned above are highly toxic to human keratocytes. Furthermore, the required treatment duration for the listed drugs is very long and may last up to six months (21, 22).

Overall, the reported and worrying lack of effective chemotherapeutic agents, have urged researchers in this field to search for novel compounds as a high priority for the treatment of *Acanthamoeba *infections. Thus, there is a raising trend to shift resources from chemical drugs to natural origin compounds (mainly isolated from plants and herbs) (23).

*Animal-based natural products*

Magainins, are defense peptides with antimicrobial activity that have been described to be secreted by the African clawed frog (*Xenopuslaevi*). These compounds cover the skin of the animal and have been reported to create an exclusive membrane-targeted mechanism of action against pathogenic agents. The reported mechanism of action involves a change in the ion conductance of membrane barriers. Magainins have been reported to be active against gram-positive and gram-negative bacteria and present anti-viral, anti-fungal and anti-parasitic effects. In the case of *Acanthamoeba*, two of the known magainins so far, MSI-103 and MSI-94 have been reported to induce amoebistatic and amoebicidal effects at concentrations from 20 to 40 µg/mL (24). Further evaluation of these compounds as anti-*Acanthamoeba* agents should be carried out against the cyst stage and also by developing *in-vivo *studies. 

*Plant-based treatments*

In recent years, many researchers working on novel therapeutic options against *Acanthamoeba* infections have focused their studies on the application of medicinal plants as a source of novel molecules with higher anti-amoebic activity and lower toxicity representing an alternative method to currently used synthetic molecules. Many plant extracts have been reported in the literature as powerful inhibitors of microbial agents including bacteria, parasites and fungi. In the case of *Acanthamoeba*, various medicinal plants and herbal extracts have been evaluated as sources of amoebicidal agents and even some of the evaluated plants have been proven to be useful for therapeutic options even* in-vivo*. Some of the test plants and herbs until now include:

*Thymus *(25)*, Satureja cuneifolia and Melissa officinalis* (26)*, Ipomoea sp., Kaempferia galanga, Canangaod orata* (27),* Trigonella Foenum Graecum *(28), *Origanum syriacum and Origanum laevigatum *(29), *Pouzolzia indica *(30),* Rubus chamaemorus, Pueraria lobata, Solidago virgaurea, Solidago graminifolia *(31),* Helianthemum lippii (L.) *(32),* garlic *(33)*, Arachis hypogaea L., Curcuma longa L. and Pancratium maritimum L *(34),* Peucedanum caucaicum, P. palimbioides, P. chryseum, P. longibracteolatum *(35),* Salvia staminea, Lnula oculus-christi (L)* (36),* Pterocaulon polystachyum *(37),* Pastinaca armenea (Fisch. &C.A.Mey.), Inula oculus-christi (L.)* (38)*, Tunisian olive *(39),* Propolis *(40)* and Buddleia cordata *(41). 

In Table 2. a list of several medicinal plants and herbs with reported amoebicidal and cysticidal effect is included and are described next:

*Thymus sipyleus subsp. Sipyleus var. sipyleus*

*In-vitro* effect of methanolic extracts of *Thymus sipyleus* subsp. *Sipyleus* var. *sipyleus* was tested against *Acanthamoeba *trophozoites (1.0 to 32 mg/mL). 

The effective activity was observed at 32 mg/mL. However, it is important to mention that this medicinal plant presented no toxicity to human keratocytes even at the highest concentration tested (32 mg/mL). A bio-guided fractionation analysis of *Thymus sipyleus* could help to find the active compounds within this plant against *Acanthamoeba* in the near future (25).

*Allium sativum (garlic)*

The anti-*Acanthamoeba* effects of the methanol extracts of *Allium sativum* (garlic) have been tested against *Acanthamoeba *trophozoites and cysts *in-vitro*. Interestingly, an amoebicidal and cysticidal activity was described for this plant species being dose and time dependent.Moreover, the tested extract was not toxic even at 3.9 mg/mL. Therefore, *Allium sativum* should be further studied in order to elucidate the novel anti-amoebic compounds presented in this plant (33).

*Ziziphus vulgaris and Trigonella foenum graecum (Fenugreek)*

Recent research carried out in our laboratories have shown that that the aqueous extracts of *Ziziphus vulgaris* and *Trigonella foenum graecum *are active against both the trophozoite and cyst stages of *Acanthamoeba*.In the case of *Trigonella foenum graecum*a concentration of 400 mg/mLwas able to eliminate trophozoites and cysts when incubated at a concentration of 750 mg/mL, after 24 h in both cases. In comparison, *Ziziphus vulgaris* aqueous extracts were able to eliminate *Acanthamoeba* trophozoites at a concentration of 200 mg/mL and cysts at 500 mg/mL, after 24 h of incubation (unpublished data). It should be mentioned that both plants did not shown toxicity when tested on cell culture at the highest evaluated concentrations. 

*Arachis hypogaea L., Curcuma longa L. and Pancratium maritimum L*

The cysticidal activity of* Arachis hypogaea L*., *Curcuma longa L*. and *Pancratium maritimum L. *was evaluated against *Acanthamoeba castellani *cysts *in vitro*. The obtained results revealed that the ethanol extract of *A. hypogaea L *had a cysticidal effect with a minimal inhibitory concentration (MIC) of 100 mg/mL in all the tested hours (24, 48, 72 h). *Curcuma longa *extracts showed MIC of 1 g/mL at 48 h and 100 mg/mL after 72 h. *Pancratium maritimum L. *also showed a MIC of 200 mg/mL after 72 h (34).

*Origanum syriacum and Origanum laevigatum*

*In vitro *evaluation of the amoebicidal activity of methanolic extracts of *Origanum syriacum* and *Origanum laevigatum* against *Acanthamoeba castellanii, *have shown that concentrations of 32 mg/mL of *Origanum syriacum *extracts, were able to eliminate trophozoites after 3 h. Moreover, incubation of cysts with extracts at the same concentration, revealed a cysticidal activity after 24 h. In the case of *O. laevigatum*, anti-trophozoite activity was observed after 72 h of incubation with extracts at a concentration of 16 mg/mL (29).

*Peucedanum caucasicum, P. palimbioides, P. chryseum and P. longibracteolatum*

Amoebicidal activity of the methanolic extracts of* Peucedanum caucasicum, P. palimbioides, P. chryseum *and* P. longibracteolatum *has been examined *in-vitro. *The obtained results in this study determined that *P. longibracteolatum* extracts presented the strongest amoebicidal effect against* Acanthamoeba*. Thus, elimination of *Acanthamoeba *trophozoites and cysts was observed between 24 and 72 h of incubation with extracts at a concentration of 32 mg/mL (35).

*Salvia staminea and Salvia caespitosa*

Amoebicidal activity of *Salvia* species has been evaluated against* Acanthamoeba castellani in-vitro*. The reported results revealed that *S. staminea *presented anti-*Acanthamoeba* effect. Moreover, the methanolic extracts of *S. staminea *were shown not be toxic to human cells even at concentrations of 16mg/mL (36).

*Satureja cuneifolia and Melissa officinalis, Ipomoea sp., Kaempferia galanga, Cananga odorata, Trigonella Foenum Graecum, Helianthemum lippii (L.), Rubus chamaemorus, Pueraria lobata, Solidago virgaurea, Solidago graminifolia, Pouzolzia indica, Pterocaulon polystachyum, Pastinaca armenea (Fisch. &C.A.Mey.), Inula oculus-christi (L.), Tunisian olive tree (Olea Europaea), Propolis and Buddleia cordata.*

*M. officinallis *has been reported to present moderate amoebicidal and cysticidal effects but *S*. *cuneifolia* presented the highest effect against trophozoites and cysts of *Acanthamoeba* (26)*. *Moreover, in another study the effect of the polar and nonpolar extracts of various plants from Southeast Asia was evaluated for their* in-vitro *amoebicidal activity against different species of *Acanthamoeba *including* A. culbertsoni, A. castellanii*, and *A. polyphaga.* The obtained results revealed that of the 200 tested plants, three species/genera (*Ipomoea sp., Kaempferia galanga, and Cananga odorata*) were active against *Acanthamoeba*. Furthermore, *Gastrochilus panduratum* extract had a lytic effect when evaluated against* A. polyphaga* and amoebistatic effects against* A. castellanii* and *A. culbertsoni *species (27).

An* in-vitro *assay developed to evaluate the amoebicidal activity of the chloroformic fraction of *Trigonella foenum graecum* has also reported this fraction to present anti-*Acanthamoebae *effects (28). In another study, four fractions of the methanolic extract of *Pouzolziaindica *were reported to present cysticidal effects (30). The amoebicidal activity of different parts of plants such as flowers, roots and leaves of *Rubus chamaemorus, Pueraria lobata, Solidago virgaurea *and* Solidago graminifolia *extracts were examined *in-vitro. *The tested extracts presented* in-vitro *and *in-vivo* against* Acanthamoeba*. Moreover, these tested extracts presented not toxic effect for the animals used in the *in-vivo *assay (31). 

The ethyl acetate and methanol extracts of *Helianthemum lippii* (L.) have been reported to present activity against *Acanthamoeba castellanii* cysts being the ethyl acetate extract, the most active extract against *Acanthamoeba* (32). Pterocaulon polystachyum (hexane fraction) extracts have been reported to eliminate 66%-70% of *Acanthamoeba *trophozoites after 48-72 h of incubation (37). In the same study, *I. oculus* showed the strongest amoebicidal effect when compared to *Pastinaca armenea* (38). 

Olive trees have also been reported to be able to inhibit the trophozoite stage of* Acanthamoeba castellanii* Neff. In this study, the activity of Olive Leaf Extracts (OLE) showed Inhibitory Concentrations of the 50% of the population (IC_50_) ranging from 8.234 μg/mL in the case of the alcoholic mixture of the Dhokkar variety, to 33.661 ± 1.398 μg/mL for the methanolic extract of the toffehi variety (39).

*Propolis* extracts have also been tested and reported to be cysticidal after incubation of *Acanthamoeba* cysts with concentrations higher than 15.62 mg/mL at 48 h or longer. Moreover, ethanolic extracts of *Propolis *have been reported to be active against *Acanthamoeba *trophozoites and cysts (40).

An *in-vitro* assay to evaluate the amoebicidal activity of the aqueous and methanolic extracts of *Buddleia cordata *against 29 strains of free-living amoebae, reported that the aqueous extract was active against 14 amoebic strains whereas the methanolic one was active against 16 strains. Nevertheless, the observed effects induced only amoebistatic effects against the tested strains. Moreover, no cysticidal activity was observed in any extract after 24 h of incubation and at concentrations up to 32 mg/ml(41). 

## Conclusion

To date, the beneficial effect of herbal medicine in many conditions such as primary dysmenorrhea, patients with diabetes and many more are studied (42).

*Acanthamoeba *keratitis is a medical challenge for most ophthalmologists. This severe corneal disease is usually treated with combination drugs such as polyhexamethylene biguanide or chlorhexidine and propamidine isethionate (15). Current therapeutic options present toxic side effects to human keratocytes and present null or low cysticidal effect (18). 

In summary, many natural products have been reported to present high anti-*Acanthamoeba* activities in the recent years. Therefore, plants extracts should be considered as a highly important and powerful source for the search of novel anti-*Acanthamoeba* compounds in the near future. 
